# Green Tea Powder Decreased Egg Weight Through Increased Liver Lipoprotein Lipase and Decreased Plasma Total Cholesterol in an Indigenous Chicken Breed

**DOI:** 10.3390/ani10030370

**Published:** 2020-02-25

**Authors:** Xingyong Chen, Kaiqin He, Congcong Wei, Wanli Yang, Zhaoyu Geng

**Affiliations:** 1 College of Animal Science and Technology, Anhui Agricultural University, Hefei 230036, China; 2 Anhui Province Key Laboratory of Local Livestock and Poultry Genetic Resource Conservation and Bio-breeding, Anhui Agricultural University, No. 130 Changjiang West Road, Hefei 230036, China

**Keywords:** egg production, green tea powder, huainan partridge chicken, lipoprotein lipase, plasma lipid

## Abstract

**Simple Summary:**

Tons of green tea powder (GTP) are produced and cast off during green tea processing. It is suggested that GTP could increase immunity and health, and so improve animal production performance. We demonstrated that one percent of GTP supplemented in the diet did not affect egg production. However, long time GTP inclusion resulted in decreased egg weight and increased feed-to-egg ratio. Combined with plasma lipid concentration, the decreased egg weight might be because of lower plasma lipid concentration, increased plasma orexin A, and liver lipoprotein lipase expression in chickens fed a diet containing GTP.

**Abstract:**

Whether or not green tea promotes egg production is unclear. Huainan partridge chickens at 20 weeks of age were divided into two groups, with one group fed a basal diet (control) and one fed a basal diet plus 10 g/kg green tea powder (GTP) for 12 weeks. Egg production (EP) and feed intake (FI) were recorded daily. Plasma lipid parameters, and apolipoprotein-B (Apo-B), 3-hydroxy-3-methylglutaryl coenzyme A reductase (HMGR), and lipoprotein lipase (LPL) expression were determined every four weeks. Egg production and FI showed no significant difference between the two groups (*p* > 0.05). Egg weight was 47.58 g in the control group, which was higher than that of the GTP group, and the feed-to-egg ratio (FCR) was 4.62 in the control group, which was lower than that of the GTP group after 12 weeks feeding. Compared with the control group, plasma orexin A (*p* < 0.05), high-density lipoprotein (HDL), apolipoprotein A (Apo A), and very high-density lipoprotein (VHDL) (*p* < 0.01, respectively) were increased. Plasma glucose (Glu), free fatty acid (FFA), apolipoprotein B (Apo B), triglyceride (TG), total cholesterol (TC) (*p* < 0.01, respectively), and low density lipoprotein (LDL) (*p* < 0.05) were decreased in the GTP group after 8 weeks feeding. The LPL expression in the liver was increased in the GTP group after 8 to 12 weeks feeding when compared to the control group (*p* < 0.05). Chickens fed GTP did not affect EP, but decreased egg weight, which might be because of lower plasma lipid concentration, increased plasma Orexin A, and liver LPL expression.

## 1. Introduction

Green tea is one of the most popular beverages worldwide and produces nearly 14,380,000 tons each year in China. Nearly 5% to 10% green tea powder is produced during green tea processing and cast off. Major components of green tea are polyphenols, including catechins (constitute about 30% of its dry weight), alkaloids, polysaccharide, etc. [1,2]. Tea polyphenols are natural antioxidants that can scavenge free radicals and protect magnum from damage [3]. Green tea could prevent dental caries and reduce cholesterol and lipid absorption in the gastrointestinal tract [4]. Koo and Noh [5] further demonstrate that green tea could lower body fat through interfering with intestinal absorption of dietary fat, cholesterol, and other lipids. Catechins, the major component in tea polyphenols, could decrease plasma and liver malondialdehyde (MDA) concentrations, plasma glucose, and total cholesterol level [6]. A meta-analysis of randomized clinical trials also suggested that green tea decreased plasma total cholesterol and low-density lipoprotein (LDL) cholesterol [1]. 

The egg is one kind of animal product that is predominantly consumed; it contains many easily absorbed nutrients, and is easy to digest [7]. However, egg production performance could be affected by many factors, including genetics, feed composition, age, etc. [8]. The consumption of eggs from indigenous breeds is widely looked for in the market [8]. The most important problem for an indigenous breed is low egg production performance. Egg production is mostly affected by follicle maturation and ovulation, which is regulated by synthesis and transportation of yolk precursors [9]. The main component for yolk precursors include yolk very low-density lipoprotein (VLDLy, more than 90%) and vitellogenin (VTG); meaning VLDLy and VTG synthesis determine the speed of follicular development [10]. As a rate-limited endogenous cholesterol synthesis enzyme, 3-hydroxy-3-methylglutaryl coenzyme A reductase (HMGR) determines egg yolk cholesterol content [11]. Apolipoprotein B (ApoB) regulates the synthesis and secretion of VLDLy. Apolipoprotein B could also increase lipoprotein lipase (LPL), binding to cells, and promoting the degradation of Very low density lipoprotein (VLDL) [12].

Wang et al. [3] suggested that adding 200 mg kg^−1^ tea polyphenols improved egg production performance in laying hens. Xia et al. mentioned that 10 g/kg green tea powder did not affect egg laying and feed conversion ratio, but a high amount of green tea powder (>20 g/kg) decreased egg production performance [13]. Our previous study also demonstrated that 10 g/kg green tea inclusion could improve meat color and Lactobacillus proliferation for broiler production [14]. 

It is well known that green tea could lower body fat in animals. However, lipids are one of the most important components in egg yolks, and determine yolk formation. Biswas et al. [15] reported that 6 g/kg of Japanese green tea powder (GTP) supplemented for laying hens tended to decrease egg weight and increase egg production rate. Thus, whether green tea powder could be used as feed additive in indigenous chicken breed during egg laying is still in doubt. The present study was conducted to evaluate the effect of 10 g/kg of green tea powder on chicken laying performance, plasma lipid content, and lipid synthesis related gene expression levels in Chinese indigenous Huainan partridge chickens.

## 2. Materials and Methods 

All the experimental protocols involving care, handling, and treatment of broilers were approved by the Institutional Animal Care and Use Committee of Anhui Agricultural University, Hefei, Anhui, China. The permission number is No. SYDW-P2018110702.

### 2.1. Birds and Experimental Design

A total of 1080 Huainan partridge hens at 18 weeks of age, with similar body weights (1.46 ± 0.13 kg), were raised in one row of battery cages. There were 30 battery cages (30 replicates, 6 tiers, and 6 cages per tier) in the row, and one hen per cage. One side of the battery cage was used as the control group and the other side was used as the experimental group. The two sides of the battery cages had equal distances to windows. The hens received 13 h light at 20 weeks old, which was extended to 16 h light at 32 weeks of age. Hens from the control group received a basal diet (without GTP), and hens from the experimental group received a basal diet plus 10 g/kg GTP, instead of bran. The feed ingredient and chemical composition are listed in Table 1. The experiment consisted of a two-week acclimation period and a 12-week collection period. During the two-week acclimation period, the diet with 10 g/kg GTP was gradually applied to hens, instead of the basal diet in one week, and another week for adaptation. Mean body weight, egg production (EP), egg weight (EW) and feed intake (FI) were calculated every 2 weeks from 20 to 32 weeks of age. The feed conversion ratio (FCR) was calculated by the formula FCR = FI/EP × EW.

At 20, 24, 28, and 32 weeks of age, thirty hens from each replicate of each group were randomly selected for blood sampling from the wing vein, and ten hens (one from three replicates) were slaughtered for liver and follicular membrane collection.

### 2.2. Blood Plasma Orexin A and Lipid Content Determination

Blood samples (2 mL) were separated by centrifugation (2500× *g* for 10 min at 4 ℃). Separated plasma were frozen for lipids and orexin A analysis within one week. Commercial enzyme-linked immunosorbent assay (ELISA) kits were used for the measurement of very high density lipoprotein (JL21659), total cholesterol (JL21710), triglyceride (JL21645), low density lipoprotein (JL15965), high density lipoprotein (JL21648), apolipoprotein B (JL45582), apolipoprotein A (JL21703), free fatty acid (JL15893), plasma glucose (JL21700), and orexin A (JL25500) by using an automatic ELISA analyzer (Rayto RT-6100). All of the kits were from Nanjing Jiancheng Bioengineering Institute (Nanjing, China).

### 2.3. Determination of mRNA Expression Level by Real-time Reverse Transcription

Liver and follicular membrane (stored in −80 ℃) were used for total RNA extraction by using a commercial kit (Omega bio-Tek Inc., GA, American) according to the manufacturer’s instructions. The quality and quantity of total RNA was determined by using Nanodrop2000 (Thermo Fisher, MA, American). After DNase treatment, 5 μg of total RNA was reverse transcribed by using RNase reverse transcriptase (Easyscript RT/RI Enzyme mix, TransGen Biotech, Beijing, China), random primers (Anchored Oligo[dT]18 Primer [0.5 μg/μL]), and random 6 mers. The mRNA expression level for each gene was determined by real-time reverse transcription according to Chen et al. [16]. β-actin was chosen as reference. The primers used for quantification were listed in Table 2. 

### 2.4. Statistical Analysis

The results from the egg production, egg weight, feed intake, feed conversion ratio, and gene expression were analyzed by two-way ANOVA, with treatment and feeding time as two variables. The results of the lipid parameters and orexin A analyses were subjected to Student *T*-tests by SAS 9.3. Data for gene expression were presented in mean ± SE, and other data were expressed as mean ± SD. 

## 3. Results

### 3.1. Laying Hens Performance 

The chicken performances during the feeding trial period were summarized in Table 3. Egg production, egg weight, and feed intake significantly increased with age (*p* < 0.01, respectively), and feed conversion ratio (FCR) significantly decreased with age (*p* < 0.01). No significant difference was observed in egg production performance between the control and GTP groups during the experimental period. Egg weight showed no significant difference between the two groups before 30 weeks of age. That is, after 10 weeks of the feeding diet, containing 10 g/kg GTP, egg weight was significantly lowered than that of the control group (*p* < 0.05). Feed intake tended to be lower in the GTP group than that of the control group at the first four weeks of feeding, and no significant difference was observed between the two groups (*p* > 0.05). Feed conversion ratio (FCR,) was relatively high before 24 weeks of age because of low egg production. A relatively higher feed intake caused a higher FCR in the control group when compared to the GTP group at 22 and 24 weeks of age (*p* < 0.05). Feed conversion ratio was higher in the GTP group than that of the control group after 10 weeks feeding (*p* < 0.05).

### 3.2. Effect of Green Tea Powder on Orexin A and Plasma Lipid Content of Huainan Partridge Chicken 

Compared with the control group, orexin A, apolipoprotein A (Apo A), and high-density lipoprotein (HDL) were significantly increased (*p* < 0.01, respectively), and glucose (Glu), free fatty acid (FFA), total cholesterol (TC), triglyceride (TG) (*p* < 0.01, respectively), and apolipoprotein-B (Apo B) (*p* < 0.05) were decreased in chickens fed diets with 10 g/kg GTP for 4 weeks. No significant difference was observed in the low-density lipoprotein (LDL) and very high-density lipoprotein (VHDL) of chickens fed a diet with GTP or not for 4 weeks. Compared with the control group, orexin A, HDL, Apo A, and VHDL significantly increased; Glu, FFA, Apo B, LDL, TG, and TC significantly decreased in chickens fed a diet with GTP for 8 or 12 weeks (Table 4).

### 3.3. Effect of Green Tea Powder on Lipid Metabolize Related Gene Expression

The expression level of genes related to lipid metabolize was listed in Table 5. The expression of Apo-B in the liver of the control group showed no significant difference at different ages (*p* > 0.05). While the Apo-B expression in the liver of the GTP group significantly increased after 4 weeks of GTP feeding, significantly decreased after 8 weeks of feeding, and kept decreasing after 12 weeks of feeding (*p* < 0.05). The expression of Apo-B in follicular membrane of the control group was significantly higher in chickens at 20 and 24 weeks of age as compared to the chickens at 28 and 32 weeks of age (*p* < 0.05). The expression of Apo-B in the follicular membrane of the GTP group significantly decreased after GTP feeding for 4, 8, or 12 weeks as compared to chickens before GTP feeding (*p* < 0.05). The expression of HMGR in the liver of the control group was higher at 20 and 24 weeks of age as compared to 28 and 32 weeks of age (*p* < 0.05). The expression of HMGR in the liver of the GTP group was decreased after GTP feeding for 4, 8, or 12 weeks as compared to chickens before GTP feeding (*p* < 0.05). The expression of HMGR in follicular membrane showed no significant difference in both control and GTP groups (*p* > 0.05). The LPL expression in the liver showed no significant difference within the control and GTP groups during the experiment (*p* > 0.05). However, the LPL expression in the liver significantly increased in chickens fed GTP for 8 to 12 weeks, as compared to control groups during the same time (*p* < 0.05). The LPL expression in the follicular membrane showed no significant difference in both control and GTP groups (*p* > 0.05).

## 4. Discussion

In our previous research, it has been demonstrated that 10 g/kg of green tea powder as a feed additive could promote intestinal health and meat quality, and did not affect the body weight of broilers [14]. Xia et al. [13] also suggested that 1% green tea powder was beneficial on egg quality from Chinese local chicken breeds, but a high amount of green tea powder (>20 g/kg) inclusion in the diet could decrease the egg weight and increase the feed-to-egg ratio. Thus, 10 g/kg of green tea powder inclusion was selected to analyze its effect on egg production performance, chicken blood parameters, and lipid synthesis related genes (Apo-B, HMGR, and LPL) expression level. 

The results of laying performance suggested that 10 g/kg of green tea powder did not affect egg laying in chickens. A digitally higher feed intake in chickens from the GTP group was observed, which might be because of increased orexin A caused by intake of green tea powder. However, Soori et al. [17] stated that green tea could reduce orexin A in overweight and obese women. Chickens before egg laying are usually under strict feed restriction to control body weight. In addition, green tea could trigger feed consumption especially under starvation. Combined with a lower palatability of diet with GTP, a digitally lower feed intake was observed in chickens fed GTP at the first four weeks. However, we also detected increased orexin A in chickens fed diets with GTP for 4 weeks, which further suggests that green tea might trigger feed consumption in laying hens after 8 weeks of feeding. 

In this research, a lower level of glucose, FFA, Apo-B, LDL, TG, and TC were detected in chickens fed diets with GTP when compared to chickens from the control group. The main components in green tea powder are tea polyphenol, caffeine, catechins, crude fiber, etc. [13]. Although some intervention research suggested no significant effects on plasma lipids after six cups of green tea per day for 4 weeks [18], there were still many studies that insisted that catechins decreased blood glucose, triglycerides, and total cholesterol content in humans and poultry [6,19]. Tea could suppress the glucose transport activity in the intestinal epithelium to lower the absorption of sugar and then reduce blood sugar levels [20]. Green tea has been demonstrated to reduce cholesterols and lipids absorption in the gastrointestinal tract [5]. It is also widely accepted that green tea consumption could reduce blood LDL and glucose levels [1,21]. A higher Apo-A, HDL, and VHDL were also observed in chickens fed diets with GTP as compared to chickens fed basal diets. Catechins from green tea could decrease the apolipoprotein B-100 (ApoB) level of human plasma in a radical reaction initiated by Cu^2+^ [22], which explained the decreased level of ApoB in chickens fed diets with GTP. In vitro experiments also demonstrated that green tea could inhibit ApoB secretion via the proteasome-independent pathway [23]. Increased HDL and apolipoprotein A (Apo A) was observed [21] in Portuguese adults who were given 1 liter of green tea per day for 4 weeks, which is in accordance with the data obtained from this experiment. During egg laying, lipogenic, and genes related to egg yolk formation in the liver were highly expressed, which caused high plasma lipid concentration in layers [24]. High plasma VLDL will then pass through the granulose basal lamina and reach the receptors located on the oocyte surface for providing yolk precursors [25]. Reduced plasma VLDL concentration might decrease egg yolk weight. The yolk weight is positively correlated with egg weight [25]. Thus, a significantly lower egg weight detected in chickens from the GTP group might be because of the decreased plasma lipid concentration. The increased feed intake and decreased egg weight caused a higher FCR (feed-to-egg ratio).

HMGR is a key rate-limiting enzyme in the synthesis of endogenous cholesterol. When dietary cholesterol absorption increased, the expression of HMGR decreased and resulted in a reduced endogenous cholesterol synthesis [11]. In this experiment, the expression of HMGR was in large quantity in the liver for the rapid development of oocytes before egg laying, and then decreased after egg laying. Under the stimulation of estrogen, large number of lipoproteins Apo-B and Apo-2 were synthesized in the liver, the secreted apolipoproteins bind with cholesterol to form VLDLy for the rapid growth of oocytes [26]. Before egg laying, plasma VLDL increased rapidly under the regulation of estrogen and the Apo-B accounts for more than 70 percent of lipoproteins [27]. Thus, it is clear that the high expression of Apo-B in liver and follicular membranes before egg laying is to promote the rapid development of follicles. As a key enzyme for regulating lipid metabolism, LPL can catalyze the hydrolysis of triglyceride to glycerol and FFA, and can remove TG-rich lipoproteins [28]. In this experiment, a significant difference was observed in the expression of LPL between the two treatment groups. It is stated that the expression of the high level of VLDL could inhibit the activity of lipoprotein lipase in laying birds; thus, allowing greater lipid transport to the ovary for egg yolk formation [24]. It is further demonstrated that higher LPL expression in chickens from the GTP group inhibited VLDL formation and yolk synthesis. 

## 5. Conclusions

In conclusion, green tea powder inclusion decreased plasma lipid concentration and increased orexin A content in Huainan partridge chickens. Green tea powder might decrease egg production after long-time inclusion through decreasing plasma TG content by increasing liver LPL expression. 

## Figures and Tables

**Table 1:** Feed composition and nutrition level.

Composition %	Group ^2^	
	CON	GTP
Soybean	22.40	22.40
Corn	66.00	66.00
Wheat bran	4.50	3.50
Lime powder	2.00	2.00
Premix ^1^	5.00	5.00
Green tea powder	0.00	1.00
Nutritional level		
Crude fat %	4.67	4.96
Total energy MJ/kg	13.07	12.99
Crude protein %	16.49	16.48
Ca % ^3^	2.00—3.20	2.00—3.20

^1^ Premix provided per kg of diet: Fe, 65 mg; Cu, 8 mg; Zn, 80 mg; Mn, 105 mg; I, 1 mg; Se, 0.3 mg; vitamin A, 9800IU; vitamin D3, 3100IU; vitamin E, 26 IU; vitamin B1, 2.5 mg; vitamin B2, 7 mg; vitamin B12, 0.018 mg; vitamin K, 2.2 mg; biotin, 0.09 mg; folic acid, 1 mg; pantothenic acid, 11 mg; niacin, 38 mg. ^2^ CON means chickens fed a basal diet as control group. GTP means chickens fed a basal diet plus 10 g/kg green tea powder. The same as Table 5. ^3^ The diet calcium was 2.0% at 20 weeks of age, and then gradually increased to 3.2% with the increased egg production.

**Table 2:** Primers for quantitative RT-PCR.

Gene	Primer Sequences (5’—3’)	Annealing Temperature ℃	Product Size (bp)
*ApoB*	CACCATCTAAAGCGTAAACCGAAC	60	196
	AAATGGGTGATTTTCAGGGTTTTT		
*HMGR*	CGTGGAATGGCAATTTTAGGTC	60	116
	CCAAAGCAGCACATGATTTCAAG		
*LPL*	GGAACAGCCAAGAAATGGAACA	60	174
	GCAGTGGTCTTGAAGAATGAGC		
*β-actin*	CTGTGCCCATCTATGAAGGCTA	60	152
	ATTTCTCTCTCGGCT-GTGGTG		

**Table 3:** Effect of dietary GTP on laying performance of Huainan partridge chickens.

Item	Treatment	Weeks of Age							SEM	*p*-Value		
		20	22	24	26	28	30	32		Age	Treatment	Interaction
EP, %												
	CON	1.91	3.10	6.85	28.99	50.43	56.62	56.49	0.98	<0.01	0.15	0.37
	GTP	1.48	3.70	9.94	29.68	51.63	55.84	55.68				
EW, g												
	CON	32.33 ^d^	33.93 ^d^	37.94 ^c^	42.71 ^b^	44.94 ^ab^	46.21 ^a^	47.58 ^a^	2.30	<0.01	0.27	<0.01
	GTP	32.82 ^d^	36.76 ^cd^	36.41 ^cd^	42.62 ^b^	44.89 ^ab^	43.84 ^b^	43.64 ^b^				
FI, g/d												
	CON	-	77.99	93.74	92.65	103.93	122.09	124.18	1.43	<0.01	0.06	0.14
	GTP	-	73.37	89.32	92.69	103.51	124.37	124.96				
FCR												
	CON	-	74.15 ^a^	36.07 ^c^	7.48 ^e^	4.58 ^g^	4.66 ^g^	4.62 ^g^	1.07	<0.01	0.023	<0.01
	GTP	-	53.94 ^b^	24.68 ^d^	7.33 ^e^	4.47 ^g^	5.08 ^f^	5.14 ^f^				

^a, b^ Different lowercase letter in the same row within the same item indicates significant difference (*p* < 0.05). EP, egg production; EW, egg weight; FI, feed intake; FCR, feed conversion ratio.

**Table 4:** Effect of Green tea powder on plasma parameters and orexin A of Huainan partridge chickens.

Treatment	Items	Week of Age			
		20	24	28	32
CON	Orexin A, pg/mL	223.3 ± 37.6	274.18 ± 50.99^B^	296.47 ± 48.68 ^b^	269.31 ± 52.57 ^B^
	Glu, mmol/L	6.86 ± 0.98	6.24 ± 0.67^A^	6.47 ± 0.79 ^A^	6.54 ± 0.93 ^A^
	FFA, μmol/L	566.1 ± 83.0	562.49 ± 56.44 ^A^	513.52 ± 76.11 ^A^	579.64 ± 74.18 ^A^
	Apo-B, μg/ml	782.9 ± 89.5	781.55 ± 116.96 ^a^	908.17 ± 91.34 ^A^	925.62 ± 112.73 ^A^
	Apo-A, μg/mL	1238.9 ± 245.3	1370.5 ± 252.12 ^B^	1197.8 ± 203.53 ^B^	1239.5 ± 139.10 ^B^
	HDL, mg/dL	101.8 ± 20.5	112.42 ± 17.49 ^B^	106.35 ± 20.48 ^B^	105.97 ± 18.30 ^B^
	LDL, mmol/L	3.54 ± 0.70	4.88 ± 0.83	4.77 ± 0.80 ^a^	5.39 ± 0.88 ^A^
	TG, nmol/L	6.35 ± 1.08	5.70 ± 0.81 ^A^	6.38 ± 0.95 ^A^	6.19 ± 0.78 ^A^
	TC, mmol/L	6.76 ± 0.87	6.62 ± 1.01 ^A^	6.42 ± 1.08 ^A^	7.00 ± 1.20 ^A^
	VHDL, mmol/L	3.15 ± 0.45	2.63 ± 0.44	2.62 ± 0.49 ^B^	2.63 ± 0.42 ^B^
GTP	Orexin A, pg/mL	232.1 ± 46.1	321.85 ± 53.15 ^A^	328.41 ± 35.08 ^a^	375.98 ± 54.35 ^A^
	Glu, mmol/L	6.76 ± 0.89	5.03 ± 0.75 ^B^	5.13 ± 0.93 ^B^	4.29 ± 0.84 ^B^
	FFA, μmol/L	559.2 ± 81.5	453.36 ± 93.85 ^B^	339.57 ± 111.24 ^B^	356.92 ± 72.63 ^B^
	Apo-B, μg/ml	735.7 ± 110.0	663.50 ± 103.76 ^b^	641.74 ± 108.14 ^B^	644.12 ± 95.95 ^B^
	Apo-A, μg/mL	1205.6 ± 290.5	1596.8 ± 295.46 ^A^	1605.2 ± 192.89 ^A^	1851.6 ± 226.76 ^A^
	HDL, mg/dL	103.7 ± 13.0	132.43 ± 17.77 ^A^	150.68 ± 16.66 ^A^	153.40 ± 12.39 ^A^
	LDL/mmol/L	388 ± 0.67	4.85 ± 0.89	4.11 ± 0.87 ^b^	3.86 ± 0.70 ^B^
	TG/nmol/L	6.53 ± 1.02	4.6 ± 0.92 ^B^	4.32 ± 0.72 ^B^	3.98 ± 0.90 ^B^
	TC/mmol/L	6.39 ± 0.79	5.59 ± 0.82 ^B^	4.05 ± 0.94 ^B^	4.32 ± 0.97 ^B^
	VHDL/mmol/L	3.45 ± 0.56	2.78 ± 0.42	3.24 ± 0.44 ^A^	3.25 ± 0.3 ^A^

^a, b^ A different lowercase letter in the same row within the same item indicates significant difference (*p* < 0.05). ^A, B^ A different uppercase letter in the same row within the same item indicates significant difference (*p* < 0.01). Glu, glucose; FFA, free fatty acid; Apo-B, apolipoprotein B; Apo-A, apolipoprotein A; HDL, high density lipoprotein; LDL, low density lipoprotein; TG, triglyceride; TC, total cholesterol; VHDL, very high-density lipoprotein.

**Table 5:** Expression of Apo-B, HMGR, and LPL in the liver and follicular membrane in Huainan partridge chickens fed GTP, or not, for different times.

Treatment	Week of Age	Apo-B		HMGR		LPL	
		Liver	Follicular Membrane	Liver	Follicular Membrane	Liver	Follicular Membrane
CON	20	1.64	5.06	3.24	1.22	1.14	1.05
	24	1.80	5.24	3.67	1.19	1.08	0.88
	28	1.18	1.56	1.02	1.00	0.43	0.56
	32	1.84	1.32	1.34	0.83	0.50	0.64
GTP	20	1.23	4.85	3.06	1.08	0.89	1.01
	24	3.61	1.71	1.48	1.19	1.34	1.17
	28	2.07	1.85	1.28	0.85	2.18	2.20
	32	1.00	1.01	1.00	1.01	2.04	2.28
SEM		0.195	0.147	0.298	0.355	0.315	0.182
*p*-value							
treatment		0.504	0.291	0.197	0.346	0.037	0.490
age		0.047	0.021	0.079	0.506	0.754	0.611
treatment × age		0.493	0.328	0.208	0.582	0.192	0.557

^a, b^ Means with no common superscripts are different within the same row (*p* < 0.05).
